# Impacting Environmental and Public Health through the Use of Dual Targeted and Tailored Asthma Educational Interventions

**DOI:** 10.1155/2015/476173

**Published:** 2015-07-09

**Authors:** Genny Carrillo, Daikwon Han, Rose L. Lucio, Yoon-Ho Seol, Betty Chong-Menard, Kenneth Smith

**Affiliations:** ^1^Department of Environmental and Occupational Health, School of Public Health, Texas A&M Health Science Center, College Station, TX 77843, USA; ^2^Department of Epidemiology and Biostatistics, School of Public Health, Texas A&M Health Science Center, 1266 TAMU, College Station, TX 77843, USA; ^3^Texas A&M Health Science Center, McAllen Campus, 2101 South McColl Road, McAllen, TX 78503, USA; ^4^Department of Health Informatics, Georgia Regents University, Augusta, GA 30912-0400, USA; ^5^Clinical Education, Respiratory Therapy Program, South Texas College, Dr. Ramiro R. Casso Nursing & Allied Health Campus, 1101 E. Vermont, McAllen, TX 78503, USA; ^6^Respiratory Therapy Services, Rio Grande Regional Hospital, 101 E. Ridge Road, McAllen, TX 78503, USA

## Abstract

Home-based asthma environmental education for parents of asthmatic children is needed since many health professionals lack the time to offer it. However, developing targeted and tailored education is important in order to address the individual needs of participants. This nonrandomized longitudinal study examined knowledge on asthma with an Asthma and Healthy Homes educational intervention training offered to parents of children from low income families who reside in the Rio Grande Valley of Texas. Eighty-nine parents received the training and pre- and posttest surveys were used to measure knowledge outcomes. A standardized assessment on asthma triggers was used to identify the different triggers each child was exposed to, and a follow-up survey was conducted 6 months after the educational intervention to identify how many parents reported household and behavior changes as a result of the training. Results showed significant changes in behavior by participants as a result of the training received. This study suggests that these behavioral changes are attributed to the dual “targeted” and “tailored” educational interventions delivered to parents which resulted in a greater understanding of how to manage asthma by eliminating asthma triggers in their respective homes.

## 1. Introduction

Asthma is a leading cause of illness and hospitalizations among children with a significant impact on their health and quality of life. More than 10 million US children under age of 18 (14%) have been diagnosed with asthma; 6.8 million children still have asthma (9%), and boys (16%) were more likely than girls (12%) to have ever been diagnosed with asthma [1].

In 2012, the National Center for Health Statistics reported the prevalence of asthma in children in the United States to be 9.3% and 11.4%, in Texas, respectively [2]. Among children in Texas, those 5–9 years of age had the highest prevalence of current asthma (10.3 percent), although not statistically significantly higher than other age groups.

Hidalgo County is located along the Texas-Mexico border within the state's Public Health Region (HSR) 11. The HSR 11 has an asthma age-adjusted hospitalization rate of 13.4% (12.5–14.3) and 14.4% (14.2–14.7) per 10,000 at the state level for children in the ages 0–17 and a large uninsured (41% in 2007) Hispanic population. Hidalgo County is the poorest county in Texas, and it is estimated that 42% of the 2,294* colonias* that exist in the Texas-Mexico border region are located in this county [3].* Colonias* are unincorporated, impoverished settlements along the Texas-Mexico border, which in many cases lack some of the most basic living necessities such as plumbing and electricity [4].

From a public health perspective, the implications of these poverty conditions translate into a disproportionate risk factors burden [5]. In the case of asthma, household environmental conditions are important because in many patients there is more than one factor or trigger for their asthma. However, optimal control is obtained when the patient, family, and healthcare team work together to prevent exposure to asthma triggers. This can be done by improving the surroundings of the asthmatic child through the elimination of environmental allergens that may aggravate their condition. These allergens may include pollen, dust mites, pet dander, mold, and cigarette smoke, a well-studied airway irritant known to cause those with asthma to have severe exacerbations [6]. Families who live in poverty along the Texas-Mexico border inhabit homes associated with unhealthy indoor environments and include houses with inappropriate roofs and floors. This increases exposure to dust mites and promotes the presence of pests such as cockroaches [7]. In many cases some homes have damaged or inappropriate pipes and plumbing facilities that promote mold development [8].

These issues represent well-documented asthma risk factors [9, 10], with a special focus on home characteristics which vary in* colonias* based upon income and location. Research shows that there is a link between health status and the home environment. These studies agree that successful asthma management involves case management and environmental adjustments [11, 12]. It has been reported that patients with asthma notice a frequent allergic and nonallergic asthma response to triggers in their environment that contribute to the frequency and severity of asthma symptoms, which could worsen their overall health condition. These triggers may be found in their residences, outdoors, places of employment, and day care settings [13]. Home-based environmental educational interventions demonstrated decreased exposure to allergens, resulting in a reduced asthma-associated morbidity [14, 15]. However, many physicians lack the time to provide this type of education to parents and children during their visits [16] and this is becoming a rarity in primary care practices [17]. It is well known that asthma attacks and hospitalizations are preventable if the patients take their medications and have the knowledge needed to avoid his/her asthma triggers. Furthermore, this knowledge cannot be gained without the use of educational interventions [18].

Tailored and targeted health communications have been used in promoting pediatric injury prevention and childhood immunizations [19, 20], in order to improve the well-being of the patient. To understand the purpose of this study, it is important to clearly define and differentiate the terms “targeted” and “tailored.” Targeting focuses on the development and implementation of a single educational intervention approach for an identified population that takes into consideration their shared characteristics [21]. Studies have found that targeting helps promote behavior change [22, 23]. By contrast, tailoring refers to “any combination of information or change strategies* intended to reach one specific person*, based on characteristics that are unique to that person, related to the outcome of interest, and have been* derived from an individual assessment*” [21].

The purpose of this study was threefold: (a) to improve the knowledge of parents of children diagnosed with asthma through a* targeted educational intervention* based on an Asthma and Healthy Homes curriculum, (b) to identify asthma triggers in the residences of participants and provide them* with tailored educational interventions*, and (c) to assess behavioral changes made by parents based on both targeted and tailored education that were provided.

## 2. Materials and Methods

This study was longitudinal and nonrandomized and targeted eighty-nine parents of children, 1–17 years of age, with a diagnosis of asthma. Pre- and posttest surveys were utilized to measure knowledge-based outcomes at baseline. Additionally, a standardized assessment on asthma triggers was applied by the* promotoras* (community health workers) to the parents of program participants in order to identify asthma triggers within their respective homes. This instrument included the collection of information relevant to the characteristics of each household which was used to tailor educational interventions at an individual level. A follow-up survey was conducted 6 months later to evaluate the degree to which participants made changes (self-reported) to their household environment based on acquired knowledge. The study was conducted in Hidalgo County, which is located in the Texas Lower Rio Grande Valley, between September 2012 and August 2014.

The eligibility criteria were a household with a child, 1–17 years of age, diagnosed with asthma and willing to participate in the two-year study. A snow-ball technique was used to identify potential study participants living in* colonias* located in Hidalgo County. The study, which was approved by the Texas A&M University Institutional Review Board (IRB), had five attritions at various times for such reasons as moving out of the city, lack of interest, the fact that the child was feeling better, and so forth. The initial asthma education was delivered as a targeted educational intervention and consisted of one 60-minute session in Spanish provided by bilingual and bicultural health professional* promotoras*. The* promotoras* were trained to obtain consent from participants, and once received they administered the Asthma and Healthy Homes survey through face-to-face interviews in English or Spanish, based on the preference of the participant, and followed up with each family six months later.* Promotoras* were also directly involved with each household and focused on the triggers and concerns identified by participants and reflected in the assessment. The data collected from these surveys was utilized to further “tailor” the educational interventions on an individual basis.

This study used a combination of target and tailor strategies. First the “target” population, parents of asthmatic children, was identified and all individuals in this population received the same educational intervention. Afterwards, each parent was administered a standardized assessment to obtain information on their specific home environment and unique circumstances related to such. Based on the outcome of these assessments, specific tailored recommendations on how to mitigate asthma triggers within the home were provided to participants. The rationale for the latter is that an individual who perceives that the education or message they are receiving responds to their particular circumstances is more likely to make a recommended behavior change. Additionally, studies have shown that exposure to tailored messages has been associated with changes in a variety of health-related behaviors [22].

## 3. Intervention

The targeted educational tool used by the promotoras was the “Asthma and Healthy Homes” curriculum, which has been certified by the Texas Department of State Health Services and used in previous studies with excellent results [24–26]. The promotoras conducted the asthma training, which was based on signs and symptoms of asthma, management of the disease, identification of common triggers, the adequate use of asthma medications, actions to take in case of an asthma attack, and basic components of an asthma action plan. All of these concepts were relevant to the targeted population as their shared characteristic was management of asthma in their children. The training lasted for 60 minutes and was provided in Spanish to all participants. As part of the same curriculum, the Seven Principles of Healthy Homes were included (how to keep a home dry, clean, well ventilated, pest-free, safe, contaminant-free, and well maintained). This curriculum was based on one developed by the National Healthy Homes Training Center and Network and focuses on improving the indoor environment and decreasing hazardous exposures within the home.

## 4. Measurements 

In order to test the knowledge acquired, a pretest assessment to measure knowledge changes among participants was applied before the one 60-minute training session. The posttest was applied three months after the education intervention was done. An asthma trigger standardized assessment was used to identify both the different triggers in each household and the physical living characteristics of participants. A follow-up survey was administered to participants by* promotoras* on a face-to-face basis 6 months after the training was completed. The purpose of the follow-up survey was to identify behavioral changes parents made as a result of the education received.

*Pre- and Posttest.* The pretest was comprised of six questions related to the participant's background and demographics and 12 true-false questions that related to asthma triggers, household chemicals, and pests. The pretest was conducted during a face-to-face interview with the parent prior to receiving the training to develop a baseline of knowledge. The posttest covered the same questions as the pretest and was applied three months after the educational intervention was completed.

## 5. Asthma Trigger Standardized Assessment

The asthma trigger standardized assessment was developed by the researchers as a checklist to identify home-based asthma triggers and preventative strategies including the following: housing structure and materials, presence of mold or water leaks, home cleaning and maintenance habits, exposure to indoor and outdoor pollution, presence of pets in the home, use of chemical irritants, smoking inside the house or car, and insect droppings. This standardized assessment was the basis for identifying and providing tailored educational interventions to individual parents based on the unique environmental circumstances in their home environments.

## 6. Follow-Up Survey

A follow-up survey was conducted six months after the first encounter with the parents to assess any behavioral changes made as a result of the Asthma and Healthy Homes' training. The follow-up survey included 33 questions regarding specific household changes and ten questions concerning changes in the home as a result of topics covered in the training, for example, water and mold, cleaning, air quality, trash, safety, hazards, paint, and lead, as well as the health of the participant's asthmatic child. This encounter was also used by the* promotoras* to reinforce the “tailored” educational concepts needed in order to address the specific needs for each family based on the information previously gathered.

## 7. Data Analysis

To estimate the impact of healthy home trainings, overall mean scores for both pre- and posttests were obtained by summing the number of correct answers for all fourteen questions (maximum score = 14), and differences in the proportion of correct answers between pre- and posttests were assessed using a paired *t*-test. Additionally, changes in numbers and percentages of pre- and posttests were compared for each individual question, and statistical significance of the difference between two frequencies was determined by the McNemar tests. Descriptive statistics were also used to summarize demographic characteristics of the respondents and frequencies of asthma triggers and follow-up surveys. Data collected at 6-month follow-up were analyzed by calculating the frequencies of the responses in different areas of household change after the training, while asthma trigger questionnaires were also examined to understand various environmental triggers at each household and physical living characteristics of participants. All analyses were conducted using SPSS version 22, and missing values were excluded from the analysis.

## 8. Results

Among the 94 families that enrolled in the study, 89 completed the program, for a retention rate of 95%. Given the longitudinal nature of the program, this retention rate is outstanding and demonstrates the promotora's skill in engaging families for a sustained period of time. Nearly all of the 89 families who completed the program reported household incomes that were less than $30,000 a year and 89% of families reported a decrease in the frequency of asthma attacks.

Table 1 shows demographic characteristics of study participants—parents of children with a diagnosis of asthma; most were female (84%), with Hispanics as majority racial/ethnic group (88%). Approximately half of the participants indicated that they have some form of medical insurance, while 45% were uninsured. About 34% reported that they had received some form of asthma education in the past.

Overall changes in knowledge mean score, obtained by summing all correctly answered questions, before and after the healthy home training were presented in Table 2. The mean score for the pretest was 12.2 (SD = 1.7), while it changed to 13.4 (SD = 0.96) after the training. The difference in the proportion of correct answers between pre- and posttests was statistically significant (*p* < 0.001). For each knowledge-based change question, percentages of correct answers before and after the healthy home training were also compared. In the pretest, the percentage of respondents who answered the questions correctly ranged between 77% and 100%, while they ranged from 87% to 100% for the posttest. In the posttest, 12 out of fourteen questions were answered correctly by more than 90% of the respondents.

We found that, out of 14 questions, six responses showed significant difference between pre- and posttest and thus are presented in Table 3. Specifically, two questions, “keeping a house well maintained is not important for the health of the inhabitants of the home” and “having fresh air circulate in home is not important,” showed more than 20% improvement in answering the question correctly after the training, while the other questions (second-hand smoke is directly linked to asthma, mold does not cause any health problems, 80% of human exposure to pesticides occurs inside the home, and second-hand smoke is directly linked to asthma) showed approximately 10% difference between pre- and posttests.

When asked about types of asthma triggers that are problematic for their children, the proportion of parents identified cigarette smoke, smoke from a campfire or wood-burning stove, strong smells, and pollen. Since it is unlikely that children's actual asthma triggers changed, these findings seem to indicate a growing awareness on the part of parents and caregivers.

Regarding asthma triggers, we found that more than half of study participants indicated that their asthma was worse around dust (83%) and that they primarily live in a dusty neighborhood (52%). The most common material for flooring in the region where the study took place is tile or vinyl due to the hot temperatures throughout the year. 82% of participants responded that they have tile or vinyl as flooring which makes cleaning it easier. However, a large percentage (81%) responded that their households have curtains or drapes which accumulate great amounts of dust. Almost half of participants have a history of allergies and 61% reported an air conditioning window unit which, in many cases, is not functioning properly and contribute to outdoor air coming inside the house. There was evidence of cockroach droppings (frass) in the households of participants and some water leakages; 44% of participants reported that their asthma is worse when aerosols or sprays are used near them and 62% when chemicals with strong odors are used in close proximity.

Table 4 shows environmental home changes reported at a follow-up survey administered to participants 6 months after the training. Almost 90% of participants indicated that they made changes as a result of the healthy homes training they received. Also included in the table are selected household changes made by the majority of the respondents: out of 24 items of environmental changes related to cleaning/maintenance, safety/hazards, ventilation, and other household items, most respondents indicated the following items: “do not allow smoking in my home (93%),” “open my windows to ventilate my home (93%),” “clean my home frequently (90%),” and “do not keep food uncovered or out in the open (90%).”

## 9. Discussion

Families and children with low socioeconomic status, minorities, and those who have either no insurance or Medicaid are at higher risk for hospitalizations and emergency department visits for asthma [27]. Children with a noncontrolled asthma visit the emergency room many times and need hospitalization for a condition that is preventable [28]. Through a multifaceted approach combining children and parental education and corresponding behavioral changes, asthma attacks can be prevented and reduced. Tobacco smoke is one of the most difficult and important triggers as it relates to asthma.

Identification of asthma triggers in each home, through the use of the standardized assessment, was needed in order to educate parents both on the environmental asthma triggers present in their home and more importantly on what to do to mitigate those triggers using the educational tools they received through the Asthma and Healthy Homes curriculum. Changes in behavior by parents of the asthmatic children who participated in the study were evident. Among nine families whose house had evidence of water damage, based on the asthma triggers checklist completed during the first home visit, five reported that they repaired the water leaks. Of four families that reported using gas stoves for heating, two indicated that they were no longer doing this during their follow-up survey. These behavioral changes are attributed to the dual “targeted” and “tailored” educational interventions delivered to parents which resulted in a greater understanding of how to manage asthma by eliminating asthma triggers in their respective homes. The education received empowered parents to better control the home environment resulting in improved health for their asthmatic child. These findings are corroborated by the literature which reports that there are improvements in asthmatic conditions when asthma triggers at home and schools are under control, since children spend most of their indoor time inside those places [29, 30].

The surveys applied in this study are not validated and they were conducted by* promotoras* trained on how to apply and fill them out, which, at some point, reduces the bias that could occur if the forms were completed by each participant. There was difficulty in reaching some parents due to disconnection of their cell phones, moving from the house, or not wanting to continue the study; this resulted in an attrition of eight parents. However, since the attrition rate was relatively low, one strength of the study was the high retention rate of participants until its completion (two years).

## 10. Conclusions

Asthma and Healthy Homes education was an important factor in improving the well-being of participants. As a result of the targeted educational intervention, study participants increased their knowledge related to asthma symptoms, case management, and how to identify triggers and exacerbating factors. In addition, due to the tailored interventions, they learned to control the symptoms of their asthmatic children by decreasing and/or eliminating their exposure to asthma triggers in their homes and thereby reduced asthma exacerbations.

In treating and preventing asthma, it is important that there is a clear understanding that, for many patients, more often than not, there may be multiple asthma triggers affecting their condition, not just one. The* promotoras* played a crucial role in this study and their work was key to the successful outcomes resulting from visits they made to each participant's home, the numerous follow-up phone calls they made, and the attention they provided to each participant. Their work resulted in an attrition rate of 5% of participants due to different reasons (moving out of town, child's asthma improved, disconnected cell phone, etc.).

This study shows that visiting households and engaging directly with parents produce an important reduction in behavioral changes regarding asthma triggers. This tailored or personal customization was well received by program participants as evidenced by changes they adopted in their respective homes. It is vital that* promotoras* use the information gained from the surveys to tailor the dialogue with the family in order to correspond directly to the needs of the family and demonstrate that their responses are being used in a way that is meaningful to them.

## Figures and Tables

**Table 1:** Demographic characteristics of study participants (*n* = 89).

Variables	*N* (%)
Gender	
Male	13 (14.6)
Female	75 (84.3)
Race/ethnicity	
Hispanic	78 (87.6)
White	8 (9.0)
Unknown	3 (3.4)
Health insurance	
Yes	44 (49.4)
No	40 (44.9)
Unknown	5 (5.6)
Asthma education in the past	
Yes	30 (33.7)
No	59 (66.3)

**Table 2:** Changes in knowledge mean score before and after the healthy home training.

All questions combined	Mean^*∗*^	SD	*p* value
Pretest score	12.18	1.71	
Posttest score	13.42	0.96	
Difference	−1.24	1.84	<0.001

^*∗*^Maximum = 14.

**Table 3:** Percentages of correct answers before and after healthy homes training: included are selected responses with significant difference between pretest and posttest.

	Pretest (%)^§^	Posttest (%)^§^
Second-hand smoke is directly linked to asthma^*∗∗*^	88.8	98.9
Mold does not cause any health problems^*∗∗*^	85.4	95.5
Having fresh air circulate in the home is not important^*∗*^	64.0	87.4
80% of human exposure to pesticides occurs inside the home^*∗∗*^	82.0	92.0
Keeping a house well maintained is not important for the health of the inhabitants of the home^*∗*^	71.9	93.3
Second-hand smoke is directly linked to asthma^*∗∗*^	88.8	98.9

^*∗*^ *p* < 0.01, ^*∗∗*^ *p* < 0.05 based on McNemar tests.

^§^Correct answers.

**Table 4:** Environmental household changes at 6-month follow-up.

	Yes (%)
In the last 4 weeks have you done any changes in your household as a result of the training you received	90
Selected changes made in the household	
Keep my home free of mold	89
Clean my home frequently	90
Open my windows to ventilate my home	93
Do not keep food uncovered or out in the open	90
Do not allow trash to accumulate in my home	85
Store medications where children cannot reach them	87
Do not allow smoking in my home	93
